# Lithium Chloride Enhances Cathepsin H Expression and BMP-4 Degradation in C3H10T1/2 Cells

**DOI:** 10.1155/2013/143742

**Published:** 2013-11-10

**Authors:** Koshi N. Kishimoto, Eiji Itoi

**Affiliations:** Department of Orthopaedic Surgery, Tohoku University School of Medicine, 1-1 Seiryo-machi, Aoba-ku, Sendai 980-8574, Japan

## Abstract

The effect of canonical Wnt/**β**-catenin signaling on chondrogenic differentiation induced by transfection of BMP4 expressing plasmid was analyzed. Lithium chloride (LiCl) which mimics canonical Wnt/**β**-catenin signaling was added to cells transfected with BMP4 expressing plasmid. Although BMP4 mRNA expression was not affected by LiCl, LiCl decreased BMP4 protein accumulation. Gene expression analysis exhibited upregulation of cathepsin H by LiCl treatment. Gene silencing of cathepsin H enhanced BMP4 protein accumulation from BMP4 expressing cells. These results suggested that cathepsin H is regulated by Wnt/**β**-catenin signaling and plays an important role in the regulation of BMP4 biological activity.

## 1. Introduction

 Bone morphogenetic proteins (BMPs) play critical roles in chondrogenic and osteogenic differentiation of mesenchymal multipotent cells [1]. The use of BMPs is a promising approach for tissue engineering of bone and cartilage. Actions of BMPs were regulated by various molecules and signaling pathways both in extracellular matrices and intracellular signal transduction. Wnt signaling is one of such pathways which regulates the cell fate in chondrogenesis and osteogenesis [2]. So far, three distinct intracellular cascades are known: canonical Wnt/*β*-catenin pathway, JNK pathway, and Wnt/Ca2+ pathway. From observations of mutant mice, deletion of canonical Wnt/*β*-catenin pathway leads to the defect of osteoblastogenesis both in endochondral and membranous ossification [3, 4]. These studies implicated that canonical Wn/*β*-catenin pathway prevents osteoblast from differentiation into chondrocyte and is required for osteogenesis. Interestingly, stabilized *β*-catenin negatively affected both osteogenesis and chondrogenesis in gain-of-function mutant study [4]. Despite these findings using mutant mice, the direct relationship between BMPs and canonical Wnt signaling is still unclear. 

 Gene transfer of BMP expressing plasmid drives recipient cells to produce BMP continuously [5]. High density micromass culture after transfection of BMP4 expressing plasmid differentiates mesenchymal C3H10T1/2 cells to chondrogenic lineage [6]. In the current study, lithium chloride (LiCl) was added on the micromass culture of BMP4 gene transfected C3H10T1/2 cells to examine the effects of Wnt/*β*-catenin signaling on the BMP4 mediated chondrogenic differentiation. LiCl inhibits glycogen synthase kinase-3*β* (GSK-3*β*) and leads to accumulation of *β*-catenin. Therefore, LiCl is known to mimic the activity of Wnt/*β*-catenin signaling [7].

From our findings in this study, we focused on cathepsin H. Cathepsin H is a member of cathepsins, which are proteases ubiquitously expressed in animal tissues. Cathepsin family includes the serine proteases cathepsins A and G, the aspartic proteases cathepsins D and E, and the lysosomal cysteine cathepsins B, C, F, H, K, L, O, S, V, X, and W [8]. Cathepsins have numerous functions in normal and pathological processes. It is widely known that cathepsin K is highly expressed in osteoclasts and plays an essential role in bone resorption [9]. Cathepsins including cathepsin H are found activated in colorectal [10], pancreatic [11], and prostate cancer [12], glioma [13], and melanoma [14]. So, cathepsins could be a therapeutic target in such malignant tumors. Cathepsin H is known to be expressed in the adult lung and process pulmonary surfactant protein [15]. From the investigation of the development of lung, Lü et al. [16] reported that cathepsin H was involved in the degradation of BMP4. We hypothesized that cathepsin H and degradation of BMP4 are related also in C3H10T1/2 cells. In the current study, we examined the gene expression and gene-silencing effects of cathepsin H.

## 2. Materials and Methods

### 2.1. Plasmid Construction and Small Interfering RNA

A 1.6 kb mouse BMP-4 cDNA is a gift from Hogan et al. [17]. It was inserted into blunted EcoRI site of the pCAGGS expression vector which was kindly provided by Miyazaki [18]. A green fluorescent protein expressing plasmid pCAGGS-EGFP was also constructed. TOPFLASH reporter plasmid containing three copies of the TCF/LEF-1-binding site and FOPFLASH containing three copies of the mutated TCF/LEF-binding site were purchased from Upstate Biotechnology (Lake Placid, NY). A sea pansy luciferase expressing plasmid with SV40, pRL-SV40, was purchased from TOYO ink. The siRNAs for cathepsin H (siCTSH: Stealth Select RNAi) and negative control siRNA (siControl: 743519) were purchased from Invitrogen. The most effective siCTSH was selected from three candidates in our preliminary experiments.

### 2.2. Cell Culture

 C3H10T1/2 clone 8 cells were obtained from RIKEN cell bank (Tsukuba, Japan) and maintained in Dulbecco's modified Eagle's medium (DMEM: Invitrogen, Carlsbad, CA) supplemented with 10% fatal bovine serum (Invitrogen), 50 U/mL penicillin, and 50 mg/mL streptomycin (Penstrep: Invitrogen), that is, growth medium. The micromass culture technique was modified from Ahrens et al. [19]. Trypsinized cells were resuspended in growth medium at a concentration of 10^7^ cells/mL. A 10 *μ*L drop of this cell suspension was placed in the center of a well in 12-well tissue culture dish (Greiner Bio-one, Kremsmuenster, Austria). The cells were allowed to adhere for 60 min and, then, the wells were filled with 1 mL of DMEM/F12 (1 : 1) supplemented with 1% fetal bovine serum (Invitrogen), 0.2% BSA (Sigma, St. Louis, MO), 50 *μ*g/mL ascorbic acid 2 phosphate (Sigma), Penstrep, and ITS+ (BD bioscience, Franklin Lakes, NJ), that is, differentiation medium. This medium was changed every 3 days.

### 2.3. In Vitro Electroporation

Before electroporation, cells were lifted with 0.25% Trypsin EDTA (Invitrogen) and dissolved in electroporation buffer [20] (75% cytosalts; 120 mM KCl, 0.15 mM CaCl_2_, 10 mM K_2_HPO_4_ pH7.6, 5 mM MgCl_2_; 25% Opti-MEM1) at a concentration of 2.5 × 10^6^ cells/mL. Plasmid solution was added into 600 *μ*L of cell suspension (1.5 × 10^6^ cells). Electric pulses were generated by CUY-21 in vitro (BEX, Tokyo, Japan) and applied through 4 mm gap cuvette. Pulse settings were 480 V, 2.5 ms, 2 pulses at the interval of 1 second. After electroporation, the cells were recovered in growth medium overnight. On the next day of electroporation, dead cells were removed by washing with phosphate buffered saline (PBS: Invitrogen) and micromass culture was started as mentioned above. The timeline of experiments was counted from the start of micromass culture in this study.

### 2.4. Alcian Blue Staining and Immunostaining of Micromass

Cultured micromass was fixed with 10% formaldehyde containing 0.1% cetylpyridinium chloride and stained by 1% Alcian blue (pH1.0). For quantification, alcian blue stain was solubilized in 4 M guanidine HCl, 50 mM Tris HCl (pH7.4), 0.1% CHAPS, and its absorbance was measured by a spectrophotometer at a wavelength of 595 nm. For immunostaining, micromass was fixed in −20°C cold ethanol. Primary antibody for BMP4 (1 : 100, sc-12721, Santa Cruz Biotechnology, Santa Cruz, CA) was added and incubated overnight at 4°C. Anti-mouse IgG conjugated with Alexa Fluoro 555 was used as secondary antibody (1 : 500). Fluorescent signal was checked by fluorescent microscope (Olympus, Tokyo, Japan).

### 2.5. Quantitative Reverse Transcription Polymerase Chain Reaction

For quantitative reverse transcription polymerase chain reaction (qRT-PCR), total RNA was isolated using RNeasy Mini kit and RNase-free DNase set (QIAGEN, West Sussex, UK) according to the manufacturer's protocol. On-column DNA digestion was routinely done. Single stranded cDNA was synthesized using High-Capacity cDNA Archive Kit (Applied Biosystems, Foster City, CA). qRT-PCR analyses were carried out with StepOnePlus and Power SYBR green master mix (Applied Biosystems). The fractional cycle number at which the fluorescence passes the threshold (Ct values) was used for quantification by using a comparative Ct method. Values of gene-of-interest (GOI) were normalized to the threshold value for GAPDH: ΔCt = Ct  (GOI) − Ct (GAPDH). The Ct value of control was used as a reference. ΔΔCt = ΔCt  (experiment) − ΔCt (control). The fold change in mRNA expression was calculated by the following formula: 2^−ΔΔCt^. Primers for RT-PCR were listed on Table 1.

### 2.6. Western Blotting

 Culture medium was subjected to SDS-PAGE with 10% acrylamide gel. Samples were blotted to a polyvinylidene difluoride membrane (Bio-Rad, Hercules, CA). The membranes were blocked with 2% nonfat dry milk for 1 hour at room temperature and incubated with anti-BMP4 primary antibody (1 : 400 Santa Cruz) at 4°C overnight. Then HRP-conjugated secondary anti-mouse IgG (1 : 1000; Santa Cruz) was applied for 2 hours at room temperature. Signals were visualized by chemiluminescence using ECL plus western blotting detection reagents (GE Healthcare, UK) with a digital luminescent image analyzer LAS-1000 (Fujifilm, Tokyo, Japan). Band intensities were analyzed by ImageJ 1.37v software program (National Institutes of Health, MD).

### 2.7. Statistics

Data were presented as means ± standard deviation of independent experiments. One-way analysis of variance (ANOVA) was performed using GraphPad Prism version 5.0 program (Graphpad software, San Diego, CA). Statistical analyses of differences between experimental groups and control were performed using Dunnett's post hoc test. Student's *t*-test was used for the analysis in the gene-silencing experiment.

## 3. Results

### 3.1. Electroporatic Transfer of BMP4 Expressing Plasmid Induced Chondrogenesis

**Figure 1:**
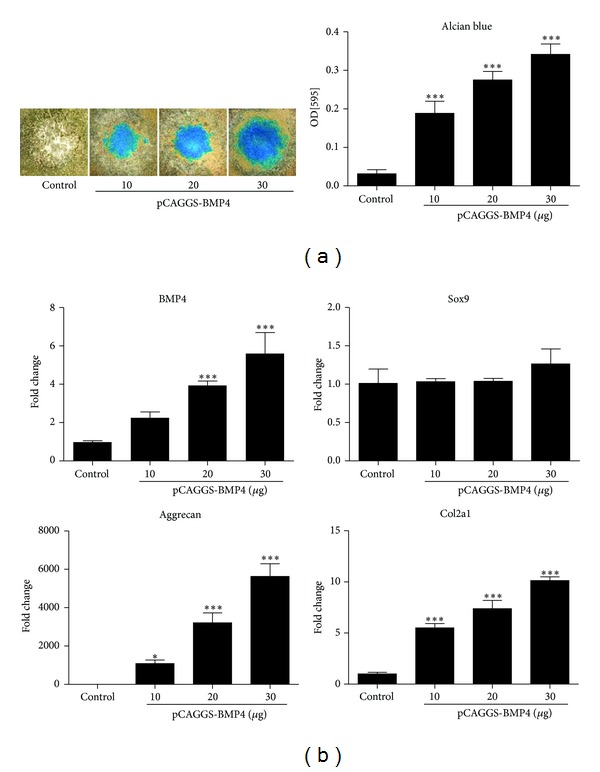
(a) Alcian blue staining of cultured micromass and quantification. C3H10T1/2 cells were transfected with BMP4 expressing plasmid, pCAGGS-BMP4. Cultured micromass was stained at day 6. The indicated amount of plasmid was supplemented in a 4 mm cuvette (600 *μ*L) at the electroporation. (b) Quantitative RT-PCR for BMP4, Sox9, Aggrecan, and Col2a1 mRNA at day 3. BMP4 mRNA expression levels and chondrogenic properties were increased in the dose dependent manner of pCAGGS-BMP4. *P < 0.05, ***P < 0.001.

To confirm the chondrogenesis, pCAGGS-BMP4 transfected C3H10T1/2 cells were cultured in micromass. From day 3, the cells metamorphosed into round shape. Alcian blue staining at day 6 exhibited positive stain. The intensity of Alcian blue staining showed dose-dependent increase depending on the amount of plasmid used at electroporation (Figure 1(a)). Gene expression profiles were analyzed by qRT-PCR at day 3. BMP4 mRNA expression increased in the dose-dependent manner of the plasmid amount. Sox9 mRNA expression was not affected by the dose of plasmid. Aggrecan and Col2a1 mRNA showed marked dose-dependent increase (Figure 1(b)).

### 3.2. The Effect of LiCl Treatment on Gene Transfected Cells

**Figure 2:**
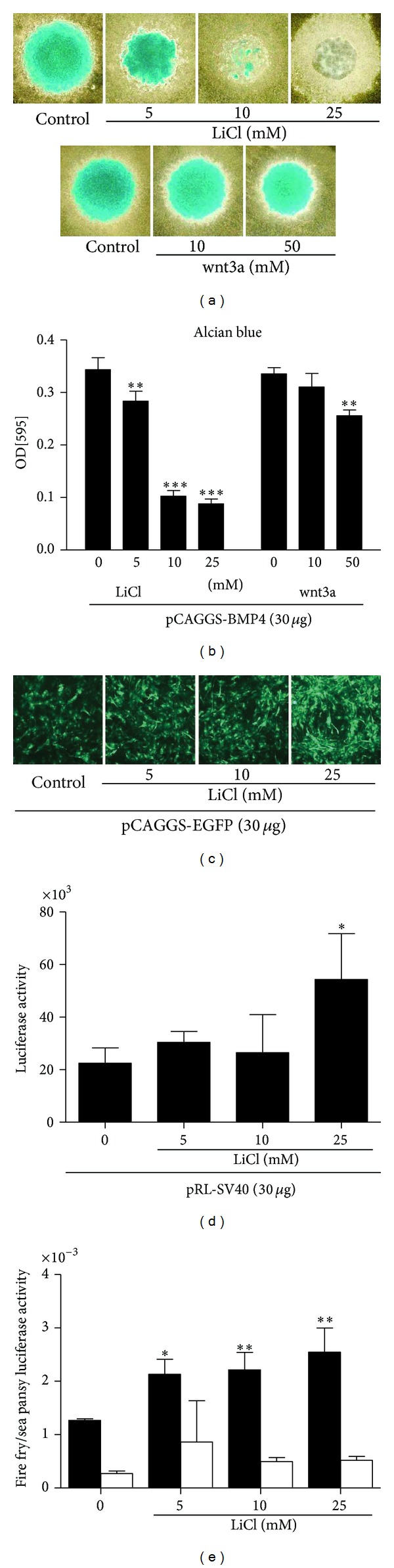
(a) Alcian blue staining of micromass. pCAGGS-BMP4 transfected C3H10T1/2 was cultured in micromass for 6 days with LiCl or wnt3a supplementation. (b) Quantitative analysis of Alcian blue staining. LiCl treatment and wnt3a supplementation suppressed chondrogenic differentiation. (c) EGFP expression of pCAGGS-EGFP (30 *μ*g/cuvette) transfected cells at day 3. LiCl did not alter EGFP expression in 5 and 10 mM and stimulated EGFP in 25 mM. (d) Sea pansy luciferase expressing gene, pRL-SV40 (30 *μ*g/cuvette), was transfected. Luciferase activity was measured at day 3. Luciferase activity was not changed in 5 and 10 mM of LiCl treatment and was higher in 25 mM. (e) Cells were cotransfected with TOPFLASH (open bar) or FOPFLASH (solid bar) (20 *μ*g/cuvette), pRL-SV40 (20 *μ*g) and pCAGGS-BMP4 (20 *μ*g). Luciferase activity in cell lysates was measured at day 3. The data was normalized to the activity of cotransfected Renilla control. LiCl treatment dose dependently increased *β*-catenin in the cells. *P < 0.05, **P < 0.01, and ***P < 0.001.

LiCl was added to the micromass culture of pCAGGS-BMP4 transfected C3H10T1/2 cells. To correct the osmolality of medium, NaCl was added up to 25 mM together with LiCl (NaCl;LiCl = 25;0, 20;5, 15;10, 25;0 mM). Alcian blue staining of micromass at day 6 was inhibited by the addition of LiCl in dose-dependent manner. The supplementation of wnt3a protein also inhibited Alcian blue staining of micromass at the concentration of 50 mM significantly (Figures 2(a) and 2(b)). To test the effect of LiCl on the expression levels of other plasmid, pCAGGS-EGFP or pRL-SV40 was transfected into C3H10T1/2 cells, and cells were cultured in micromass under the same concentrations of LiCl. The expression of EGFP was similar to that of the control in 5 and 10 mM of LiCl treatment and higher in the 25 mM LiCl treatment (Figure 2(c)). The luciferase assay showed similar tendencies to the EGFP expression (Figure 2(d)).

### 3.3. LiCl Treatment Activated *β*-Catenin/TCF Pathway

To confirm the activation of canonical Wnt signaling in the LiCl treated micromass, TOPFLASH or FOPFLASH (20 *μ*g/cuvette), pRL-SV40 (20 *μ*g) and pCAGGS-BMP4 (20 *μ*g), was cotransfected by electroporation. After a 3-day culture in micromass with or without LiCl, luciferase activity of cell lysate was assessed. The promoter activity of TOPFLASH was enhanced by addition of LiCl (Figure 2(e)).

### 3.4. LiCl Treatment Suppressed Chondrogenic Differentiation of C3H10T1/2

**Figure 3:**
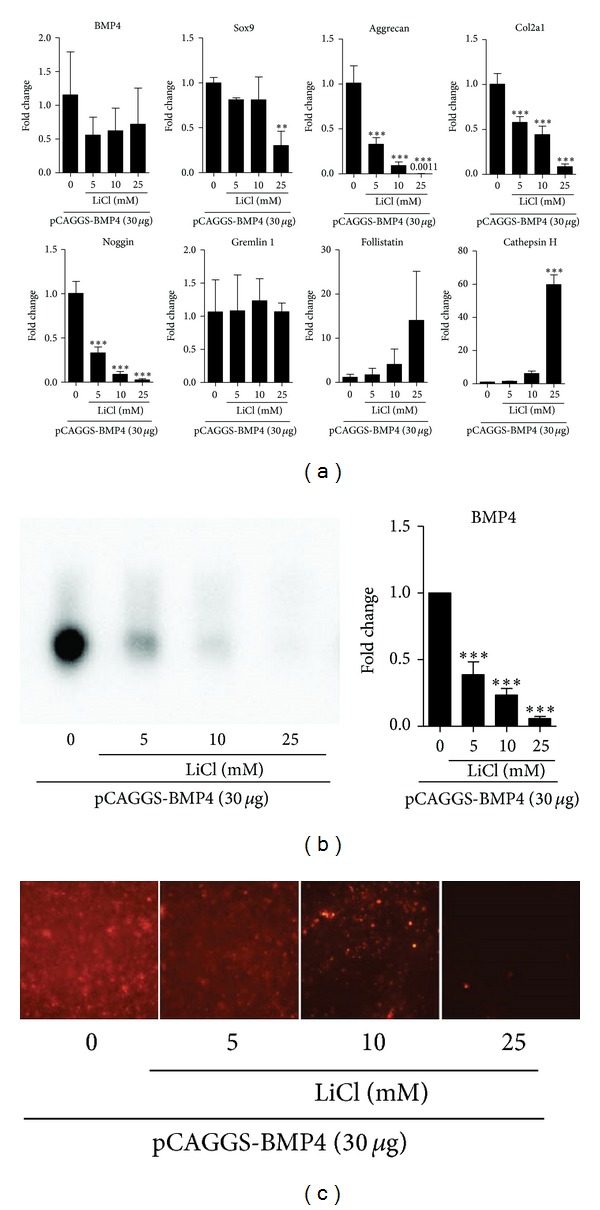
(a) Gene expression profiles of pCAGGS-BMP4 transfected cells cultured in micromass at day 3. (b) Western blot analysis of culture medium for BMP4 at day 3. (c) Immunostaining of micromass for BMP4 at day 3. BMP4 gene expression levels were not significantly altered by LiCl. However, BMP4 protein accumulation in both culture medium and cells was dose dependently decreased by LiCl. Aggrecan and Col2a1 mRNA expression was decreased by LiCl. BMP antagonist, noggin, gremlin 1 and follistatin showed different responses to LiCl. The mRNA expression of cathepsin H was up-regulated by LiCl. **P < 0.01, ***P < 0.001.

The gene expression profiles of C3H10T1/2 cells transfected with pCAGGS-BMP4 (30 *μ*g/cuvette) were analyzed at day 3 (Figure 3(a)). The levels of BMP4 mRNA did not show significant difference by LiCl treatment. Sox9 mRNA expression was significantly suppressed at 25 mM LiCl treatment. Aggrecan and Col2a1 gene were dose-dependently inhibited by LiCl. The expression levels of antagonists against BMPs were also investigated. Noggin mRNA was dose-dependently suppressed. Gremlin 1 expression were not affected by LiCl. The levels of follistatin were increased in dose-dependent manner. The expression levels of cathepsin H was increased in a dose-dependent manner of LiCl.

### 3.5. LiCl Treatment Remarkably Suppressed BMP4 Protein Accumulation

The conditioned medium used in the micromass culture of BMP4 transfected cells was subjected to western blot analysis (Figure 3(b)). LiCl treatment dose dependently reduced the accumulation of BMP4 protein in the culture medium. Localization of BMP4 protein was assessed by immunocytochemical staining of micromass (Figure 3(c)). BMP4 protein in the cells and extracellular matrix was also reduced by LiCl treatment in dose dependent manner. 

### 3.6. Gene Silencing of Cathepsin H Stimulated the Protein Expression of BMP4

**Figure 4:**
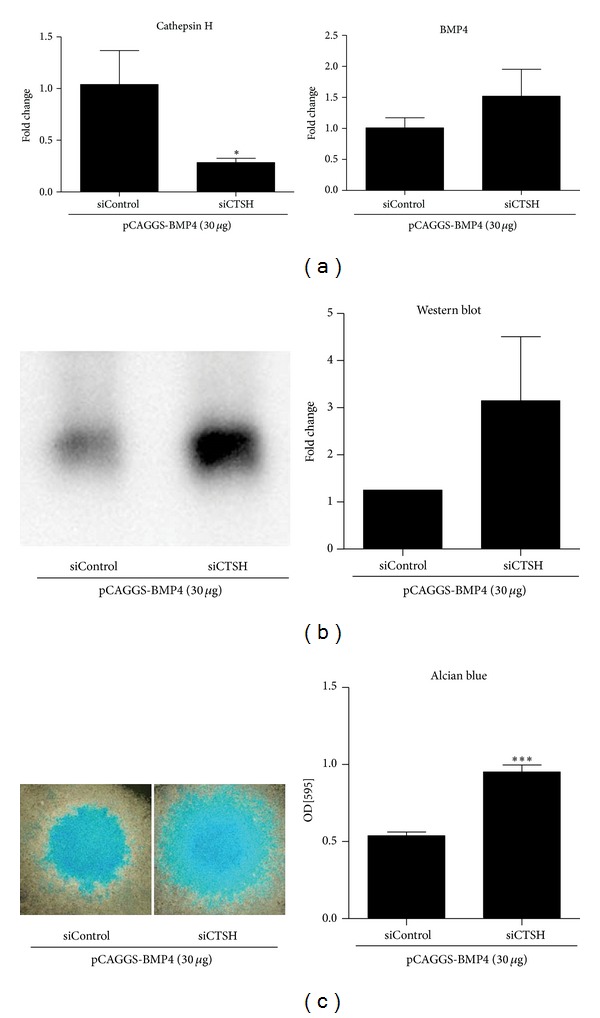
Gene silencing of cathepsin H was carried out by electroporatic gene transfer of small interfering RNA. siCTSH targeting cathepsin H or siControl (300 pmol/cuvette each) was transfected with pCAGGS-BMP4 (30 *μ*g/cuvette). (a) Quantitative RT-PCR at day 3. Cathepsin H mRNA expression was inhibited significantly. (b) Western blot analysis for BMP4 in the culture medium at day 3 showed marked increase of BMP4. (c) Alcian blue staining of micromass showed significant increase by gene silencing of cathepsin H. *P < 0.05, ***P < 0.001.

RNA interference for cathepsin H was carried out by electroporatic transfer of small interfering RNA molecule. After cotransfection of siCTSH or siControl (300 pmol/cuvette) with pCAGGS-BMP4 (30 *μ*g/cuvette) by electroporation, cells were cultured in micromass. qRT-PCR at day 3 showed suppression of cathepsin H mRNA expression. Cathepsin H gene silencing slightly increased the expression level of BMP4 by 1.5 ± 0.4-folds (Figure 4(a)). Western blotting for BMP4 showed that cathepsin H gene silencing remarkably increased BMP4 protein expression by 2.9 ± 1.4-folds (Figure 4(b)). Alcian blue staining of micromass at day 6 showed significant increase in siCTSH transfected cells (Figure 3(c)).

## 4. Discussion

 In the current study, BMP4 expressing cells were treated with LiCl which stabilize *β*-catenin and activate Wnt/*β*-catenin signaling. LiCl treatment did not affect the mRNA expression from transfected BMP4 expressing plasmid. However, LiCl dramatically reduced the accumulation of BMP4 protein both in conditioned medium and micromass. This discrepancy between gene expression and protein accumulation could be attributed to the degradation of BMP4 protein. Cathepsin H which is known to be involved in the degradation of BMP4 protein in the developmental stage of lung [16] showed increase in the dose-dependent manner of LiCl. Gene silencing of cathepsin H enhanced the protein accumulation of BMP4 from expressing plasmid. These results confirmed that cathepsin H is involved in the degradation of BMP4 protein in the C3H10T1/2 and its expression was regulated by Wnt/*β*-catenin signaling. The current study suggested that LiCl treatment which mimics Wnt/*β*-catenin signaling controls the biological activity of BMP4 through the degradation process by cathepsin H, for the first time.

 Cathepsin H is a protease which has both endopeptidase and aminopeptidase activities [21]. From the spatiotemporal expression pattern in the developing lung, Lü et al. [16] identified the degradation of BMP4 by cathepsin H as a posttranslational system which controls the bioavailability of BMP4. Pharmacological inhibitors of cathepsin H increased BMP4 accumulation and disrupted the lung morphogenesis. In the current study, our result of BMP4 protein and siRNA analyses ensured that degradation of BMP4 by cathepsin H occurs in other situations than lung development. From our observation by immunostaining and western blotting for BMP4, BMP4 protein was diminished by LiCl not only in conditioned medium but also in the cells. These results suggest that degradation of BMP4 occurred in the lysosomes or endosomes of the cells. However, it could not be denied that cathepsin H degrades BMP4 extracellularly. Our preliminary experiment (data not shown) and Lü's data [16] failed to demonstrate BMP degradation by simple in vitro mixture of BMP4 and cathepsin H proteins. This may imply that cathepsin H requires another cofactor or specific environment to exhibit BMP4 degradation activity. Lysosomal cathepsins require slightly acidic environment, such as that found in the lysosomes [8]. So, it is likely that cathepsin H degrades BMP4 in the lysosomes or endosomes. Ishihara et al. [22] analyzed global gene expression using microarray at the fracture site adenovirally infected by BMP genes in an equine model. The expression of cathepsin H was greater in BMP-2 and -6 treated fracture callus than that control. This may imply cathepsin H plays a certain role in the activity of BMPs at fracture site. Only a few researches have shed light on the degradation of BMPs. There are many issues awaiting investigation, such as degradation of other members of TGF-*β* superfamily, BMP degradation ability of other members of cathepsin family, and the details of degradation process.

 BMP4 protein is synthesized as an inactive precursor and undergoes proteolytic cleavage by subtilisin-like proprotein convertase at the specific site to be activated [23, 24]. So far, no evidence has been shown that cathepsins are involved in the cleavage of BMP precursors. According to suppliers information, the antibody for BMP4 used in the current study reacts with both BMP4 precursor and mature BMP4. In our immunocytostaining for BMP4, LiCl treatment also suppressed the BMP4 accumulation in the cells. Cathepsin H is a lysosomal cysteine protease. Therefore, cathepsin H seemed to degrade both BMP4 precursors and mature BMP4 in cytoplasm before its extracellular secretion.

 Noggin is a well-known antagonist of BMPs. Noggin binds to BMPs with high affinity and blocks interaction between BMPs and their receptors [25]. BMPs including BMP-4 induce the expression of noggin [26]. This phenomenon suggests that there exists a negative feedback loop to limit the activities of BMPs. The suppression of noggin expression along with the reduction of BMP4 accumulation in our observation is consistent with previous findings. Gremlin expression was not influenced by LiCl in our data. Sun et al. suggested that gremlin is also expressed in developing lung with BMP4 [27]. Interestingly, gremlin interacts with BMP4 precursor intracellularly and efficiently inhibits the activity of BMP4. Cathepsin H is also expressed in developing lung and inhibits BMP4. There may exist an interplay between gremlin and cathepsin H. Follistatin also binds BMP4 and inhibits its activity extracellularly. On the contrary to noggin, it is known that follistatin expression is downregulated by BMP4 in chondrocytes [28]. The decrease of BMP4 accumulation by LiCl seemed to stimulate the expression of follistatin by the reduction of inhibition in our experiments.

 In the current study, LiCl was used to mimic the action of canonical Wnt/*β*-catenin signaling. The presence of LiCl inhibits glycogen synthase kinase-3*β* (GSK-3*β*). Intracellular *β*-catenin undergoes digestion via ubiquitin proteasome system when the receptor frizzled and coreceptor LRP were not bound with Wnt ligand. The inhibition of GSK-3*β* blocks this digestion and increases the accumulation of intracellular *β*-catenin [7]. LiCl inhibited the chondrogenic differentiation of C3H10T1/2 micromass culture treated with BMP2 protein supplementation [29]. This data suggested that Wnt/*β*-catenin signaling directly inhibits chondrogenesis. In our experiments, Wnt/*β*-catenin signaling synergistically inhibits chondrogenesis of C3H10T1/2 cells through both the degradation of BMP4 and direct inhibition by Wnt/*β*-catenin signaling.

 Gene silencing of cathepsin H in BMP4 expressing cells exhibited greater accumulation of BMP4 protein in the absence of LiCl. This result without mimicking Wnt/*β*-catenin signaling may suggest that catalytic processes through cathepsin H are active at a certain level in C3H10T1/2. Although this activity could vary according to types of cells and conditions, the inhibition of cathepsin H has a potential to enhance the effects of BMPs in the gene-based tissue engineering of bone and cartilage.

## 5. Conclusion

 In conclusion, upregulation of canonical wnt/*β*-catenin signaling mimicked by LiCl caused discrepancy between mRNA and protein expression of BMP4. Cathepsin H, a cysteine protease which degrades BMP4 mRNA expression, was also upregulated by LiCl treatment. Gene silencing of cathepsin H enhanced BMP4 accumulation from BMP4 expressing cells. Wnt/*β*-catenin signaling may regulate the biological activity of BMP4 through the regulation of cathepsin H.

## Figures and Tables

**Table 1:** Primers for RT-PCR.

Gene	Accession number	Forward (5′-3′)	Reverse (5′-3′)
*BMP4 *	NM_007554.1	gaggagtttccatcacgaaga	gctctgccgaggagatca
*Sox9 *	NM_011448.2	cagcaagactctgggcaag	tccacgaagggtctcttctc
*aggrecan *	NM_007424.1	ccagcctacaccccagtg	gagggtgggaagccatgt
*Col2a1 *	NM_031163.2	agtaccggagctcgaggag	gatcacccttggcaccag
*Noggin *	NM_008711	cggccagcactatctacaca	gttcgatgaggtccaccaag
*Gremlin 1 *	NM_011824	gaggacccacggaagtga	cctcagctgttggcagtagg
*Follistatin *	NM_008046	tggattagcctatgagggaaag	tggaatcccataggcatttt
*Cathepsin H *	NM_007801.1	gaggaagattcaagcccaca	aaaactggttcaacgccatt

## References

[1] Reddi AH (2000). Morphogenesis and tissue engineering of bone and cartilage: inductive signals, stem cells, and biomimetic biomaterials. *Tissue Engineering*.

[2] Kolpakova E, Olsen BR (2005). Wnt/*β*-catenin—a canonical tale of cell-fate choice in the vertebrate skeleton. *Developmental Cell*.

[3] Day TF, Guo X, Garrett-Beal L, Yang Y (2005). Wnt/*β*-catenin signaling in mesenchymal progenitors controls osteoblast and chondrocyte differentiation during vertebrate skeletogenesis. *Developmental Cell*.

[4] Hill TP, Später D, Taketo MM, Birchmeier W, Hartmann C (2005). Canonical Wnt/*β*-catenin signaling prevents osteoblasts from differentiating into chondrocytes. *Developmental Cell*.

[5] Kishimoto KN, Watanabe YY, Nakamura HH, Kokubun SS (2002). Ectopic bone formation by electroporatic transfer of bone morphogenetic protein-4 gene. *Bone*.

[6] Kishimoto KN, Watanabe Y, Nakamura H (2009). Bone formation by BMP gene transfection. *Electroporation and Sonoporation in Developmental Biology*.

[7] Klein PS, Melton DA (1996). A molecular mechanism for the effect of lithium on development. *Proceedings of the National Academy of Sciences of the United States of America*.

[8] Turk V, Stoka V, Vasiljeva O (2012). Cysteine cathepsins: from structure, function and regulation to new frontiers. *Biochimica et Biophysica Acta*.

[9] Costa AG, Cusano NE, Silva BC, Cremers S, Bilezikian JP (2011). Cathepsin K: its skeletal actions and role as a therapeutic target in osteoporosis. *Nature Reviews Rheumatology*.

[10] Kuester D, Lippert H, Roessner A, Krueger S (2008). The cathepsin family and their role in colorectal cancer. *Pathology Research and Practice*.

[11] Sulpizio S, Franceschini N, Piattelli A, di Sebastiano P, Innocenti P, Selvaggi F (2012). Cathepsins and pancreatic cancer: the 2012 update. *Pancreatology*.

[12] Jevnikar Z, Rojnik M, Jamnik P, Doljak B, Fonovic UP, Kos J (2013). Cathepsin H mediates the processing of talin and regulates migration of prostate cancer cells. *Journal of Biological Chemistry*.

[13] Sivaparvathi M, Sawaya R, Gokaslan ZL, Chintala SK, Rao JS (1996). Expression and the role of cathepsin H in human glioma progression and invasion. *Cancer Letters*.

[14] Frohlich E, Schlagenhauff B, Mohrle M, Weber E, Klessen C, Rassner G (2001). Activity, expression, and transcription rate of the cathepsins B, D, H, and L in cutaneous malignant melanoma. *Cancer*.

[15] Ueno T, Linder S, Na C-L, Rice WR, Johansson J, Weaver TE (2004). Processing of pulmonary surfactant protein B by napsin and cathepsin H. *Journal of Biological Chemistry*.

[16] Lü J, Qian J, Keppler D, Cardoso WV (2007). Cathespin H is an Fgf10 target involved in Bmp4 degradation during lung branching morphogenesis. *Journal of Biological Chemistry*.

[17] Jones CM, Lyons KM, Hogan BLM (1991). Involvement of Bone Morphogenetic Protein-4 (BMP-4) and Vgr-1 in morphogenesis and neurogenesis in the mouse. *Development*.

[18] Miyazaki J-I, Takaki S, Araki K (1989). Expression vector system based on the chicken *β*-actin promoter directs efficient production of interleukin-5. *Gene*.

[19] Ahrens PB, Solursh M, Reiter RS (1977). Stage-related capacity for limb chondrogenesis in cell culture. *Developmental Biology*.

[20] van den Hoff MJB, Moorman FFM, Lamers WH (1992). Electroporation in ‘intracellular’ buffer increases cell survival. *Nucleic Acids Research*.

[21] Turk V, Turk B, Turk D (2001). Lysosomal cysteine proteases: facts and opportunities. *The EMBO Journal*.

[22] Ishihara A, Shields KM, Litsky AS (2008). Osteogenic gene regulation and relative acceleration of healing by adenoviral-mediated transfer of human BMP-2 or -6 in equine osteotomy and ostectomy models. *Journal of Orthopaedic Research*.

[23] Constam DB, Robertson EJ (1999). Regulation of bone morphogenetic protein activity by pro domains and proprotein convertases. *Journal of Cell Biology*.

[24] Cui Y, Jean F, Thomas G, Christian JL (1998). BMP-4 is proteolytically activated by furin and/or PC6 during vertebrate embryonic development. *The EMBO Journal*.

[25] Zimmerman LB, de Jesús-Escobar JM, Harland RM (1996). The Spemann organizer signal noggin binds and inactivates bone morphogenetic protein 4. *Cell*.

[26] Gazzerro E, Gangji V, Canalis E (1998). Bone morphogenetic proteins induce the expression of noggin, which limits their activity in cultured rat osteoblasts. *The Journal of Clinical Investigation*.

[27] Sun J, Zhuang F-F, Mullersman JE (2006). BMP4 activation and secretion are negatively regulated by an intracellular Gremlin-BMP4 interaction. *Journal of Biological Chemistry*.

[28] Tardif G, Hum D, Pelletier J-P, Boileau C, Ranger P, Martel-Pelletier J (2004). Differential gene expression and regulation of the bone morphogenetic protein antagonists follistatin and gremlin in normal and osteoarthritic human chondrocytes and synovial fibroblasts. *Arthritis and Rheumatism*.

[29] Fischer L, Boland G, Tuan RS (2002). Wnt signaling during BMP-2 stimulation of mesenchymal chondrogenesis. *Journal of Cellular Biochemistry*.

